# How Diet and Physical Activity Modulate Gut Microbiota: Evidence, and Perspectives

**DOI:** 10.3390/nu14122456

**Published:** 2022-06-14

**Authors:** Daniela Campaniello, Maria Rosaria Corbo, Milena Sinigaglia, Barbara Speranza, Angela Racioppo, Clelia Altieri, Antonio Bevilacqua

**Affiliations:** Department of Agriculture, Food, Natural Resources and Engineering (DAFNE), University of Foggia, 71122 Foggia, Italy; daniela.campaniello@unifg.it (D.C.); mariarosaria.corbo@unifg.it (M.R.C.); milena.sinigaglia@unifg.it (M.S.); barbara.speranza@unifg.it (B.S.); angela.racioppo@unifg.it (A.R.); clelia.altieri@unifg.it (C.A.)

**Keywords:** gut microbiota, Mediterranean diet, ketogenic diet, physical exercise, endurance fitness

## Abstract

Gut microbiota plays a significant role in the maintenance of physiological homeostasis, contributing to human health. Nevertheless, some factors (sex, age, lifestyle, physical activity, drug-based therapies, diet, etc.) affect its composition and functionality, linked to pathologies and immunological diseases. Concerning diet, it interacts with microorganisms, leading to beneficial or detrimental outcomes for the health of host. On the other hand, physical activity is known to be useful for preventing and, sometimes, treating several diseases of cardiovascular, neuroendocrine, respiratory, and muscular systems. This paper focuses on diet and physical activity presenting the current knowledge about how different diets (Western, ketogenic, vegan, gluten free, Mediterranean) as well as different types of exercise (intensive, endurance, aerobic) could shape gut microbiota.

## 1. Introduction

Nowadays, in developed countries, men reach the age of 79 and women up to 84 years old. Thus, adults over the age of 65 are projected to represent 20–25% of the total population in Europe and the United States by 2030. Therefore, the population is aging, and modern societies must face this problem, worsened by the fact that elderly subjects often have pathologies associated with a series of comorbidities [1].

Having a healthy lifestyle is a goal that everyone should aim for; having a good sleep, a balanced and healthy diet, physical activity, free time to devote to hobbies, and living in healthy environments are some of the key factors to achieving a healthy lifestyle. An active lifestyle, including daily physical exercise, is considered an optimal method to preserve health in all stages of life. The reduction of body weight, counteraction of blood hypertension and dyslipidemia, or attenuation of insulin resistance are only some of the benefits to prevent undesirable events, such as cardiovascular problems and metabolic disorders [1]. On the other hand, a balanced diet providing the energy and nutrition we need is also crucial in promoting our well-being state. Both factors can influence the health of the human organism as they can influence the intestinal microbiota, defined as the “assemblage of living microorganisms presents in a defined environment” [2]. For example, Mika et al. [3] conducted a study on young rats who experienced physical activity voluntarily every day. The researchers observed that, thanks to physical exercise, rats developed a more advantageous microbial structure, with the expansion of some intestinal probiotic bacterial species. Furthermore, compared to the groups of sedentary rats, adults, and regularly active adults, young rats had: (i) changes in the qualitative composition of gut-microbiota, with a modulating effect on the taxa able to affect fat composition in the body; (ii) abundance of beneficial microbial species; (iii) increase in butyrate, which is linked to some epigenetic processes.

Diet and physical activity, together with other environmental factors (work activity, socio-economic status, etc.), act during a lifetime, affecting the type of the individual’s intestinal microbiota, which changes in relation to the different age and in relation to the characteristics of the individual [4]. Considering the growing interest towards “microbiota” topic, this article focuses on diet and physical activity as factors able to drive changes in the quali-quantitative composition of gut microbiota. Thus, this paper is composed of two parts: a first section focused on how different diets can modulate the microbiota and a second section reporting an overview on microbiota and physical activity.

## 2. How Diet Affect Microbiota Composition: An Overview

Several factors, such as genotype, sex, age, immune status, and various environmental factors, cause an inter-individual variability of human gut microbiota. Amongst these factors, dietary habits play a fundamental role in shaping the gut microbiota composition [5]. A famous sentence of Hippocrates is: “Let food be the medicine and medicine be the food” since he considered foods one of the main tools that doctors can use. Effectively, the main function of foods is to satisfy the hunger needs of each person; on the other hand, foods are also essential for sustaining human growth, reproduction, and health. Therefore, nutrient intake is important for our survival and well-being, but it is also crucial for modulating the symbiotic microbial communities living in the intestine, that is gut microbiota. Microbiota is composed of Bacteroidetes, Firmicutes, Proteobacteria, Actinobacteria, Verrucomicrobia, Cyanobacteria, and Fusobacteria, but Bacteroidetes and Firmicutes are the most abundant and their activity has been particularly studied [6].

The source, quality, and type of food shape gut microbiota, influencing its composition and function and impacting on host-microbes’ interactions. In fact, gut microbiota composition is different between individuals, and, as reported by Leeming et al. [7], diet is responsible of at least 50% variability in mice and 20% in humans, thus stressing the importance of some dietary strategies to counteract diseases through positive quali-quantitative changes in gut microbiota. Therefore, it is essential to study how diet can impact on gut microbiota and which nutritional components can better shape it, promoting a healthy microbial community.

In the past, some researchers studied oral microbiota from skeleton teeth of people over the various epochs. The results showed that the most notable changes in human gut microbiota happened both during the transit from the hunter-gatherer paleolithic era to the farming neolithic era, characterized by diet rich in carbohydrates, and at the beginning of the industrialised period, whose main sign was a diet rich in processed flour and sugar [8]. Other studies focused on the changes resulting from the agricultural era. In fact, before the development of agriculture and animal farming, humans ate wild plants, animal meats, and minimally processed foods. Then, the initial domestication of plants and animals led to changes in the nutrient composition of these foods. In the pre-agricultural diet, foods as cereals, sugars and refined vegetable oils, dairy foods, alcohol, salt, and fatty domesticated meats did not exist. Nowadays, they represent the primary constituents of the post-agricultural, and typical Western diet [9]. The importance of some dietary constituents has been clearly reported by Yatsunenko et al. [10], who documented variations in gut microbiota between Americans and Africans. The United States population has mainly a low-fiber diet while African and South American people consume large quantities of plant-based polysaccharides. Comparing the gut microbiome of these populations, the authors observed that microbiota of United States population was far less diverse than African and South American people.

According to the foods we supply to our organism, different types of microorganisms are fed. This led to the identification of three intestinal microbiota models (or enterotypes), different for the most abundant microbial species present. Enterotype 1, which has *Bacteroides* as the most abundant genus, is typical of the industrialised countries where diet is based on high-fat diet and low fiber intake, and where refined and/or industrialised foods, red meats, and dairy products are consumed in large quantities. Enterotype 2, with *Prevotella* as one of the most abundant components, is typical of less industrialized countries where diet is generally based on the consumption of fibers rather than of meat and dairy products [11]. Finally, enterotype 3, with *Ruminococcus,* a mucin-degrading bacterium as the most important constituent, is the least common enterotype.

The prevalence of a specific enterotype depends also on long-term dietary habits, because high-fat and protein diet favours enterotypes 1 and 3, while a diet rich in carbohydrates supports the rise of enterotype 2, but short-term habits could also exert an effect.

David et al. [12] reported that gut microbiota can be modified by short-term dietary changes, but that these changes persist only for a few days. In fact, gut microbiota resists some external inputs, including extensive dietary changes, thus maintaining its own composition [7].

### 2.1. Western Diet

Many studies suggest that lifestyle changes, including urbanisation, dietary behaviours, low physical activity, excessive use of antibiotics, and improved hygiene practices have affected quali-quantitative traits of gut microbiota, thus contributing to the emergence of some pathological conditions of modern civilisation [9]. All these aspects are part of the Western lifestyle, although diet is the leading factor able to extensively modulate gut microbiota [12]. The Western diet is considered responsible of the intestinal dysbiosis that stimulates local inflammation, causing an increase of intestinal permeability through the proliferation of pro-inflammatory species [13], even if the exact mechanism is not clear. High consumption of red meat, saturated fats, and sugars, and in general of processed foods, as well as a low intake of fibers, are the main traits of Western diet. This type of diet causes a reduction of some bacterial species associated with anti-inflammatories conditions, such as *Akkermansia muciniphila, Faecalibacterium prausnitzii, Roseburia* spp., *Eubacterium* spp., and *Clostridium* cluster XIVa and IV [14,15], and the ability to produce beneficial metabolites is consequently reduced in the human gut.

The role of fatty acids in the modulation of gut microbiota has been recognized. Dietary fatty acids are divided into three main categories: saturated fatty acids (SFA), monounsaturated fatty acids (MUFA), and polyunsaturated fatty acids (PUFA). Essential PUFAs are represented by two important families: ω-6 and ω-3. Numerous studies revealed that a high-fat diet rich in safflower oil (that contains ω-6 PUFA) reduces the populations of *Bacteroides* while augmenting the populations of Firmicutes, Actinobacteria and Proteobacteria [16]. In vitro studies have estimated the effect of PUFAs on the adhesion properties and growth of different *Lactobacillus* strains (*Lacticaseibacillus rhamnosus* GG, *Lacticaseibacillus casei* Shirota and *Lactobacillus delbrueckii* subsp. *bulgaricus*) obtaining different results. High amounts of PUFA inhibited both adhesion to mucus and growth, while small amounts of gamma-linolenic acid and arachidonic acid exerted a positive effect on *Lcb. casei* Shirota [17,18]. Costantini et al. [19] have dedicated an interesting review on the impact of ω-3 PUFAs on the intestinal microbiota and the main effects can be summarized as follows: (i) when ω-3 PUFAs are supplemented to adults, gut microbiota showed a reduction in *Faecalibacterium*, as well as an increase in Bacteroidetes and *Lachnospiraceae* family (butyrate-producing bacteria) linked to higher amounts of anti-inflammatory compounds, such as short-chain fatty acids (SCFAs), (ii) ω-3 PUFAs could affect the gut–brain axis, acting through gut microbiota composition; (iii) the interaction ω-3 fatty acids-animal gut microbiota promotes the maintenance of the intestinal wall integrity.

Concerning ω-6 PUFA, a high intake of these compound caused a reduction in Bifidobacteria [19,20]. Similarly, high intake of MUFA was associated with lower concentration of *Bifidobacterium* spp. and moderately higher numbers of *Bacteroides* spp. [20].

A high fat diet could cause endotoxin (or lipopolysaccharide) production by intestinal microbiota thus, providing an optimal condition for the gram-negative bacteria proliferation, such as *Enterobacteriaceae*. This increase in endotoxin levels can result in inflammation and alteration in the gut microbiota composition by influencing the intestinal permeability and favouring obesity [21]. Moreover, increased gut permeability is associated with a reduction in *Bifidobacterium* spp. Bifidobacteria generally exert a positive effect, because they can reduce LPS levels and improve gut barrier function [22]. Some studies on the effect of high-fat dietary pattern demonstrated that a regular consumption of red meat can cause a quantitative increase in *Bacteroides* [23], while the frequent use of milk (containing saturated fats) has shown the capacity to stimulate the growth of delta-Proteobacteria, mainly *Bilophila wadsworthia* [24]. In both human and mouse models, a diet with a high-fat content enhanced the incidence of colonic inflammation. This result seems to be induced by the secretion of bile acid, mainly taurine, stimulated by fatty food. Such a phenomenon promotes the growth of sulphite-reducing pathobionts such as *B. wadsworthia* [24].

### 2.2. Ketogenic Diet

The ketogenic diet is a diet with a low intake in carbohydrates (5–10% of the total daily caloric intake) aimed at increasing the metabolism of fats with a reduction in glucose levels to favour, on the other hand, the production of ketone bodies (3-hydroxybutyrate, acetate and acetoacetate) through hepatic ketogenesis, hence its name. An increase in ketones results in: (i) energy source for colonocytes (colon cells), (ii) increase in anti-inflammatory and antioxidant activity, (iii) immune regulation, (iv) intestinal mobility and barrier functionality, (v) cell growth and differentiation, (vi) ionic absorption, and the (vii) prevention of distal ulcers, Crohn’s disease, and colon cancers [25].

The ketogenic diet has been used as a therapeutic treatment for several neurological disorders, such as Alzheimer’s disease, Parkinson’s disease, depression, and epilepsy [26]. Recent studies in mice have revealed that the ketogenic diet has a protective effect due to the modulation of gut microbiota, if followed for short periods [27,28]. Olson et al. [28] showed that ketogenic diet, in mice, increased the abundance of *Akkermansia* and *Parabacteriodes*. Paoli et al. [25] analyzed nine studies concerning the impact of ketogenic diet on the intestinal bacterial component, and found specific alterations based on the condition of the subjects and/or the single study, that is: (i) in 12 epileptic children, an increase in *Escherichia coli* and a decrease in *Bifidobacterium*, *Eubacterium rectale*, and genus *Dialister* were observed; (ii) in 20 patients affected by refractory epilepsy, a ketogenic diet caused a significant rise of Bacteroidetes and a lowering of Firmicutes and Actinobacteria, while in another study (14 patients and 30 healthy infants, also suffering a refractory epilepsy) a significant rise of Bacteroidetes (*Bacteroides*, *Prevotella*), *Bifidobacterium* and a reduction of Proteobacteria (*Cronobacter, Escherichia, Salmonella, Vibrio*) were detected; (iii) Concerning autism spectrum disorders, gut microbiota experienced a higher abundance of *Enterobacteriaceae* and an increased ratio of Firmicutes*/Bacteroides*; while *A. muciniphila* decreased in both colon and faeces. Instead, in healthy subjects (mouse models), the most important changes were linked to a rise of *A. muciniphila* and *Lactobacillus* and a lowering of *Desulfovibrio*, *Turicinabacter*, and overall bacterial diversity.

The results are contradictory. They show that keto-diet affects gut microbiota, but it is not clear which are the taxa mostly affected. More recently, Rondanelli et al. [29] evaluated the effects of very low-calorie diets (VLCDs; with high-protein, low carbohydrate ≤ 30%, a maximum intake of 800 kcal/day), very low-calorie ketogenic diet (VLCKDs; carbohydrates < 70 g/day, intake <800 kcal/day) and very low carbohydrate diets (VLCarbDs) on the relative abundances of taxa constituting gut microbiota.

The results showed that in obese patients the Bacteroidetes/Firmicutes ratio was strongly affected, causing a reduction in short-chain fatty acid production. VLCKD-induced weight loss, a reduction in *Eu. rectale*, *Roseburia*, and an increase in *Akkermansia* along with *Christensenellaceae*.

### 2.3. Vegan Diet

The vegan diet only includes foods of plant origin, in particular a high intake of fruit and vegetables and a low intake of saturated fats, which means that it does not include products of animal origin [30]. A characteristic of plant species is that they contain phytochemicals, comprising carotenoids and polyphenols. Generally, these substances do not provide energy in term of kcal, but they are important for some metabolisms thus exerting a positive effect on human health [31,32]. However, if a vegan diet is not properly balanced, an intake of some compounds (fatty acids, proteins, vitamins, and minerals) below recommended levels could occur [33]. It is not clear if the vegan diet is linked and related to a particular model of gut microbiota, but it is well known the ratio P/B (*Prevotella* vs. *Bacteroides*) could be increased by a higher intake of fibers, while a Western type-diet could negatively affect it, in combination with environmental, cultural, and genetic factors [34,35]. Generally, *Prevotella* species are more abundant in individuals following a diet with a high intake of plant-based compounds, like in Asia, Africa, and South American societies, while *Bacteroides* is the main trait of populations of Western societies, with high levels of animal proteins, and saturated fats [36,37]. Fibers could affect both quantitative (density) and qualitative traits of gut microbiota (type and number of bacterial species). In fact, high intakes of indigestible carbohydrate (wheat bran and whole grain) generally cause a rise of *Lactobacillus* spp. and *Bifidobacterium* spp., while whole grain barley and resistant starch are positively linked to *Ruminococcus* spp., *Eu. rectale*, and *Roseburia* spp. and negatively associated to some taxa of Firmicutes phylum like *Clostridium* and *Enterococcus* species [38,39]. The increase of lactobacilli and bifidobacteria levels is very important for the resistance of gut to pathobionts because they inhibit colonization and growth of bacterial pathogens thanks to their saccharolytic metabolism [39,40].

It is known that fermentable dietary fibers serve as substrates for intestinal bacteria metabolism, and the final compounds of their pathways are SCFAs [41], such as acetate, propionate, and butyrate, which are used as energy substrates by gut epithelial cells [42,43]. They are directly or indirect linked to some positive pathways, like acidification of colon, reduction of plasma level of cholesterol, increase of glucose tolerance and insulin sensitivity, bioactivity towards *Enterobacteriaceae* pathogens (like *Salmonella* spp., adherent-invasive *E. coli*), absorption of water and sodium, and anti-inflammatory effects; moreover, they are energy sources for colonic epithelial cells, contribute to cancer prevention by inhibiting cell inhibition proliferation, and induce fat oxidation, thus preventing obesity [44,45,46]. Another key class for a positive modulation of gut microbiota comprises polyphenols (flavonoids, stilbenes, lignans, phenolic acids, and secoiridoids) at high levels in vegan diets. They enter small intestine and hereby contribute to a positive modulation of *Lactobacillus* spp. and *Bifidobacterium* spp. [39,47].

Some studies have reported a positive modulation of soy or tea isoflavones on gut microbiota, while the assumption of wild blueberries was described as the leading factor for a significant increase of *Bifidobacterium* and *Lactobacillus* species [48]. *Clostridium histolyticum* and *Clostridium perfringens* were reduced after the consumption of fruit, seed, tea, and wine polyphenols [38], as well as the *Enterobacteriaceae* family in diets rich in proanthocyanidin-rich extract from grape seeds, while *Bifidobacterium* spp. were enhanced [49].

### 2.4. Gluten Free Diet

The gluten-free diet (GFD) could significantly influence gut microbiota. A study showed that one month of GFD in healthy volunteers decreased populations of *Lactobacillus* and *Bifidobacterium* while increasing *E. coli* and *Enterobacteriaceae* generally linked to episodes of bacteremia [50]. In addition, Bonder et al. [51] reported that a GFD in healthy volunteers decreased the abundance of *Roseburia* and increased the abundance of *Victivallaceae* and *Clostridiaceae* [51]. These studies demonstrated how a GFD, that is considered a valid therapy for celiac patients, is often associated with health issues and low intakes of some nutrients.

Although a GFD is necessary for diseased patients, there is an increasing trend towards this kind of diet due to the fake news that it is healthful for the general population. Many people adopt this diet without medical support by ignoring that GFD generally causes an increased food cost, a potential decrease in fiber, mineral, and vitamin consumption (calcium, magnesium, zinc, vitamin B12, folate, and vitamin D), and potentially increased exposure to dietary hydrogenated and saturated fatty acids and arsenic. Thus, it would be convenient that, under medical supervision, celiac, wheat allergy, or nonceliac gluten sensitivity patients, adopt a GFD; while for individuals insisting on a GFD but without the pathologies above reported, nutritional counseling is recommended to reduce potential risks [52].

### 2.5. Mediterranean Diet

Mediterranean diet (MD) is generally based on non-refined grains, legumes, fresh vegetables, fruits, and nuts, while the consumption of milk and yogurt, eggs, white meat, fish and seafood, and high fat dairy products (few times per week), red meat, and ethanol (a few times per month) is reduced. EVO (extra-virgin olive oil) is the main source of lipids [53].

MD modulates the composition and functionality of human gut microbiota with a well-defined imprinting in the microbiome and metabolome and a reduced risk of illness if compared to Western diet. For example, a study showed how MD for one year in twenty obese men decreased *Prevotella* and increased *Oscillospira* and *Roseburia* [54]. *Roseburia* produces butyrate, thus contributing to microbial homeostasis and to the reduction of inflammation [55] while *Oscillospira* is linked to weight balance and healthiness [56]. A long-term MD increased the relative abundance of *Parabacteroides distasonis* [54], which is an interesting species able to control/inhibit obesity-induced tumor formation in mice [57]. Moreover, a two-year MD positively acted on *Bacteroides* and *Prevotella* and on some saccharolytic genera, including *Roseburia*, *Faecalibacterium*, and *Ruminococcus* [58]. A study reported that MD, in a cohort of 153 Italian individuals, increased the levels of fecal SCFAs and the relative abundances of Firmicutes and Bacteroidetes, while a low adherence to the MD was the leading factor for an increased level of urinary TMAO (TriMethylAmine N-Oxide) and of *Ruminococcus* [35], and, as reported by Lombardo et al. [59], high blood TMAO levels might be associated with heart disease, atherosclerosis, diabetes, and cancer. Another study showed how the consumption of MD in 74 Spanish volunteers determined an increase of the levels of *Clostridium* cluster XVIa and *F. prausnitzii* [60]. Among the species of *Clostridium* cluster XVIa, there is *Blautia coccoides*, which produces butyrate and secondary bile acid, and it is involved in the formation of T-regulatory cells [61,62], while *F. prausnitzii* is as strong producer of butyrate and has very high anti-inflammatory traits [63].

## 3. Microbiota and Physical Activity

It is well known that physical activity takes care of our cardiovascular health and promotes our psychological well-being due to the stimulation of endorphin production. Regular physical activity, in fact, ameliorates the tolerance to glucose and the symptoms of anxiety, stress, and depression and lowers the risk of type 2-diabetes, hypercholesterolemia, hypertension, heart disease, various cancers, and obesity and prevents osteoporosis. Furthermore, physical activity could interact with intestinal microbiota, modulating its composition and playing a positive role in homeostasis and energy regulation [64]. Low-intensity exercise can affect the gastrointestinal tract (GIT) by reducing the transient evacuation time and therefore the contact time between pathogens and the gastrointestinal mucus layer. As a result, exercise appears to have protective effects, reducing the risk of colon cancer, diverticulosis, and inflammatory bowel disease [64].

How physical exercise modulates the intestinal microbiota is depending on several factors such as training intensity, environment, and diet. Some papers have recently shown that physical activity is a potential factor favouring the biodiversity of the intestinal microbial ecosystem both in qualitative and quantitative terms, suggesting that the beneficial effects of physical exercise on gastrointestinal function, mood, and other brain functions, could be mediated by modifications of the microbiota. Unfortunately, the mode of action by which physical activity determines these changes is not yet clear; probably, it includes various interrelated factors and pathways, such as (i) changes in the bile acids profile which could exert antimicrobial activity and/or select certain bacterial species; (ii) increased production of immunoglobulins A (IgA) linked to the resistance to colonisation by specific microorganisms, (iii) increased production of SCFA, (iv) suppression of Toll-like receptor 4 (TLR4) signaling pathways, which can reduce serum lipopolysaccharides (LPS) levels; (v) release of myokines from muscle fiber cells, (vi) improvement of body composition, (vii) maintenance of glycemic homeostasis, (viii) reduction of intestinal transit time, and (ix) activation of the hypothalamic-pituitary-adrenal HPA axis and successive production of hormones caused by physical stress [65].

### 3.1. Animal Models

Several studies conducted on animals have shown that training alters the composition and functional capacity of the gut microbiota. Mailing et al. [66] report that the first evidence on the beneficial effects of exercises on the gastrointestinal environment were shown by Matsumoto et al. [67] who evaluated how running affected on the cecal microbiota. They tested 14 male Wistar rats divided in two groups for five weeks: the control group included sedentary individuals, while the exercise group could use a wheel in their cage. Voluntary running exercise in rats involved a change in the composition of the microbiota, an increase in the concentration of n-butyrate, and an increase in the diameter and weight of the cecum. In detail, concerning composition, butyrate-producing bacteria were detected, and strictly connected to an increase in the concentration of n-butyrate (8.14 ± 1.36 mmol/g of cecal contents in the exercise group versus 4.87 ± 0.41 mmol/g of cecal contents in the control group). The diameter of the cecum was about 1.5-fold larger in the exercise group than in the control.

Moreover, in obese rats, exercise modulated microbiota. In high fat diet-induced obesity mice, exercise affected their gut microbiota composition producing a microbial composition similar to lean mice and regulated changes at phylum-level. In fact, a high fat diet caused an increase in some constituents of Firmicutes (*Lactobacillaceae*, *Lachnospiraceae*, and *Ruminococcaceae*) and a decrease in one family of Bacteroidetes (S24–7). Exercise prevented dietary changes [68]. Similarly, Campbell et al. [69] evaluated the effect of exercise on gut microbiota in 36 male mice fed by a normal or high-fat diet for 12 weeks and randomly assigned to exercise or sedentary groups. Exercise determined an increase of *F. prausnitzii* in training mice, able to protect the digestive tract by producing butyrate and lowering the oxygen tension in the lumen. Furthermore, exercise altered intestinal villi morphology, induced by a high-fat diet.

Understanding how exercise alters the gut microbiota of mice is difficult because there are many variables involved (diet, species/strain, animal age and exercise method) and sometimes the results are contradictory. For example, some studies suggest that exercise increases the Firmicutes/Bacteroidetes ratio, while other studies suggest that exercise reduces it [66].

### 3.2. Intensity of Physical Exercise and Intestinal Microbiota

Fecal microbiota characteristics, dietary intake and body composition were compared in 15 healthy sedentary men (as controls), 15 bodybuilders, and 15 long distance runners to study the long-term effects of a specific type of exercise combined to different model diets, on gut microbiota [70]. The type of exercise was associated with the dietary patterns of athletes (i.e., bodybuilders: high-protein, high-fat, low-carb/dietary fiber diet; distance runners: low-carb and dietary fiber diet). Although the type of activity did not affect alpha diversity of the gut microbiota, differences were noted in the relative abundance of different bacteria. For example, in bodybuilders, the genera *Clostridium*, *Eisenbergiella*, *Faecalibacterium*, *Haemophilus*, and *Sutterella* were over-represented, while *Bifidobacterium* and *Parasutterella* were less abundant. Probiotic species, such as *Bifidobacterium adolescentis*, *Bifidobacterium longum*, *Latilactobacillus sakei*, and SCFA-producing microorganisms (*Blautia wexlerae*, *Eubacterium hallii*), were lower in bodybuilders and higher in controls. A negative correlation between protein intake–diversity (in distance runners) and fat intake–bifidobacteria (in bodybuilders) was also observed. These differences could be due to the nutritional status of the athletes (i.e., insufficient carbohydrates and dietary fiber; higher fat).

### 3.3. Endurance Physical Exercise and Intestinal Microbiota

Endurance exercise can be defined as cardiovascular exercise performed for an extended period (long-term physical activity). Running, cross-country skiing, cycling, aerobic exercise, or swimming are examples of endurance exercises through which athletes expose their bodies to extreme physiological conditions that disrupt the inner body’s homeostasis, involving organs and the system’s normal function. With these changes, the body responds through the synthesis of proteins, hormone release, and shifts in fluid and metabolic balance and with an improvement of mechanical, metabolic, neuromuscular, and contractile functions [71]. The effect of endurance exercise was evaluated studying fecal metabolites and microbiota of 20 amateur runners before and after a half marathon race using metabolomics and sequencing analysis of 16S ribosomal RNA [72]. Differences concerning the microbial population, before and after runs, were observed. At the phylum level, Lentisphaerae and Acidobacteria, whose functions in the human gut are unknown, were detected after the run. At the family level, an increase in *Coriobacteriaceae* and *Succinivibrionaceae* was detected. *Coriobateriaceae* (phylum Actinobacteria) are involved in the metabolism of bile salts and steroid hormones, as well as in the activation of dietary polyphenols in the human intestine. They have been positively correlated with 15 metabolites, indicating that *Coriobacteriaceae* metabolism may be a potential mechanism underlying the role of exercise in disease prevention and improvement of health outcomes. In fact, the increase in these metabolites running could promote a good composition of gut microbiota and the enhancement of positive metabolic pathways. At the genus level, half marathon running seemed to lower the levels of *Ezakiella*, *Romboutsia*, and *Actinobacillus*, while increasing *Coprococcus* and *Ruminococcus bicirculans*. *Actinobacillus* spp. is linked to some animal diseases (actinomycosis in cattle, potent septicemia in the newborn foal and human periodontal disease). Thus, the inhibition of this potential pathogen was correlated to an anti-inflammatory effect of exercise.

Taniguchi et al. [73] investigated whether endurance exercises modulate gut microbiota in elderly subjects: 33 elderly Japanese men (aged 62–76 years) participated in a 5-week endurance exercise program. The monitored parameters were CRF (cardiorespiratory fitness), nutritional intake via a questionnaire, some parameters relating to blood tests, blood pressure, cardio-ankle vascular index, collection of fecal samples, and extraction of intestinal microbial DNA. Analyses on microbial composition showed that the relative abundance of *Clostridium difficile* decreased significantly, while that of *Oscillospira* increased during exercise compared with the control period. Another study focused on the effects of 12 weeks of exercise in healthy elderly women [74], showing an increase in the levels of *Bacteroides* in subjects who completed aerobic exercise. It is widely recognized that lower *Bacteroides* levels are associated with a higher prevalence of obesity and metabolic syndrome and that *Bacteroides* species may help suppressing metabolic dysfunction.

Scheiman et al. [75] recruited athletes who had to run the Boston Marathon, and a series of ‘sedentary’ subjects (control) to identify gut bacteria associated with athletic performance and recovery states. Sequencing of 16S ribosomal DNA was conducted on daily fecal samples collected up to one week before and one week after the marathon event. Significant differences in *Veillonella* abundance were observed after the marathon, with higher levels in runners. *Veillonella* species metabolize lactate into SCFAs such as acetate and propionate via the methylmalonyl-CoA pathway. Thus, intestinal colonisation of *Veillonella* can increase the Cori cycle through which lactate is converted into SCFAs that could aid performance. Furthermore, Scheiman et al. [75] isolated a strain of *Veillonella atypica* in fecal sample from one of the marathon runners and inoculated it in gnotobiotic mice, finding a significant reduction in inflammatory cytokines after exercise compared to control. They also showed an increased ratio of conversion of lactate into propionate.

Keohane et al. [76] observed how ultra-endurance exercise alters the gut microbiome of four well-trained ultra-tough male athletes subjected to prolonged, high-intensity ocean rowing, describing changes in microbial diversity, abundance, and metabolic capacity. Stool samples were obtained from athletes for metagenomic sequencing to record the microbial community structure and related functional gene profiles before the competition, mid-competition, after the competition, and three months after the competition. The most important changes were as follows: (i) a higher abundance of butyrate-producing species and species associated with improved metabolic health and improved insulin sensitivity; (ii) higher levels of species responsible of the biosynthesis of healthy amino and fatty acids (e.g., L-isoleucine and L-lysine). These positive amino acids are linked to hematopoiesis, which in turn can increase oxygen carrying capacity and cardiorespiratory fitness. Many of the adaptations in microbial community structure and meta-proteomics persisted after three months.

### 3.4. Microbiota, Physical Exercise, and Aerobic Power

Considering the importance of cardiorespiratory fitness to safeguard the cardiovascular and pulmonary system some studies evaluated the correlation with this type of sport and gut microbiota composition. Estaki et al. [77] tried to correlate the microbiota composition and its metabolic capacity with cardiorespiratory fitness. This study tested healthy adults with varying cardiorespiratory fitness levels (*n* = 39) and found that a higher diversity of the microbiota, as well as the concentration of butyrate-producing taxa, was positively correlated with cardiorespiratory fitness, regardless of the diet of individuals [77]. Another study conducted by Durk et al. [78] on young healthy individuals (*n* = 20 men, *n* = 17 women) with varying cardiorespiratory fitness levels showed that a higher Firmicutes/Bacteroidetes ratio was significantly correlated with maximal oxygen consumption expressed as VO_2max_ (i.e., the maximum volume of oxygen consumed in one minute measured with treadmill).

In a study on premenopausal women, cardiorespiratory fitness was associated with the composition of the gut microbiota, regardless of age and carbohydrate or fat intake. Participants with low VO_2max_ had low *Bacteroides* levels, but higher *Eu. rectale* and *Clostridium coccoides* levels than the high VO_2max_ group [79].

Morita et al. [74] studied healthy elderly women (32 sedentary women, >65 years) for a 12-week non-randomized comparative study. They were divided into two groups (trunk muscle training, TM, or aerobic training, AE). Fecal samples were analyzed before and after the training period to determine the composition of the gut microbiota. The 12-week aerobic exercise training increased the relative abundance of intestinal *Bacteroides*, along with an improvement in cardiorespiratory fitness. On the other hand, training of the trunk muscles did not change the composition of the intestinal microbiota, although it improved cardiorespiratory activity.

### 3.5. Microbiota, Exercise, and Inflammatory State

Exercise causes microbiome changes that can induce inflammatory, immune, and oxidative responses leading to improvements of metabolic disorders [80]. Exercise modifies gene expression of intraepithelial lymphocytes, downregulating pro-inflammatory cytokines and upregulating anti-inflammatory cytokines and antioxidant enzymes [66]. Likewise, it can affect the integrity of the intestinal mucus layer, which plays an important role in preventing microbes from adhering to the intestinal epithelium and acts as an important barrier for some mucosal associated bacteria.

Motiani et al. [81] recruited 26 sedentary middle-aged subjects, nine of whom were prediabetic and 17 with T2D (type 2 diabetes). Participants followed a two-week-training (three times/week), using two training programs, SIT (sprint interval training) and MICT (moderate-intensity continuous training). The authors monitored how short-term training affects intestinal insulin-stimulated glucose uptake (GU), fatty acids uptake (FAU), intestinal microbiota composition, and metabolic endotoxemia (lipopolysaccharide binding protein, LBP), which is an intestinal inflammatory marker. Physical exercise reduces intestinal inflammation and modulates the profiles of the intestinal microbiota in insulin-resistant subjects. Both SIT and MICT reduced endotoxemia by decreasing the intestinal inflammatory marker (LBP), Firmicutes/Bacteroidetes ratio (obesity), the genus *Clostridium* (immune response), and *Blautia* (inflammation). Conversely, Bacteroidetes (protection against obesity) increased. Thus, short-term exercise training improved the gut microbiota profiles and reduced endotoxemia.

## 4. Conclusions and Future Perspectives

Every healthy human is characterized by a unique gut microbiota, and a healthy gut microbiota composition is different for everyone. Moreover, gut microbiota constitutes a changing ecosystem influenced by many factors such as stress, unbalanced diet, antibiotic use and more. In this article, we have mainly focused attention on the influence of nutrition and physical activity in the modulation of the microbiota, and consequently on the state of human health. Regarding human well-being, there is a close connection between gut microbiota, nutrition, and physical exercise and there is not a perfect formula that can guarantee the maintenance of the state of health, but many variables that can enter the game.

Diet is key factor for microbiota modulation, mainly for long term habits and status, which could affect health and well-being by quali-quantitative changes in the relative abundances of some taxa and in the amounts of some metabolites. Eating habits, including fruits and vegetables, dietary fibers, MUFAs, and PUFAs, promote gut microbiota diversity and functionality. Also, nutrients, as shown in the Supplementary Material S1 and in Tables S1, S2 and S3 could differently modulate microbiota.

Dietary patterns have significantly changed since the origin of mankind, as hominids’ diet included raw vegetables and low amounts of proteins of animal origin, while the modern diet has high lipid and energy intakes and is based on processed and refined foods. Therefore, understanding the role of gut microbiota in the link between diet is pivotal for a personalized nutrition, able to counteract and/or to prevent ‘Western-associated diseases’, especially metabolic-related diseases for which microbiota quantity and quality can represent a crucial aspect.

Concerning exercise, through a search in the literature, we found that the current literary corpus stresses that physical activity is an important environmental factor, capable of inducing changes in the intestinal microbial composition both from a qualitative and quantitative point of view, favoring a healthier state of the individual.

Exercise can have several effects. The most important are the following:

1. enrichment of the diversity of the microbiota;
2. improvement of the *Bacteroides*/Firmicutes ratio, which could potentially contribute to promoting weight loss, the reduction of diseases associated with obesity and gastrointestinal disorders;
3. stimulate the proliferation of bacterial species that can modulate mucosal immunity and improve barrier functions, with a consequent reduction in the incidence of obesity and metabolic diseases;
4. stimulate bacterial species capable of producing substances that have a protective action against gastrointestinal disorders and colon cancer (such as SCFAs).

In particular, the variables of intensity and duration of exercise could be key parameters for a significant change. Further studies are necessary to fully understand the mechanisms that determine the changes in the composition and functions of the microflora caused by exercise and all their related effects.

The gut microbiome has been shown to mediate the effect of diet and exercise, making it relevant to athletes’ health and performance. Hence, it is important that future research implements an appropriate protocol able to discriminate among diet and exercise effects. Furthermore, further studies including the combined analysis of metabolomic and metagenomic data could open new perspectives for studying the correlation between diet, sport, and health.

**Figure 1:**
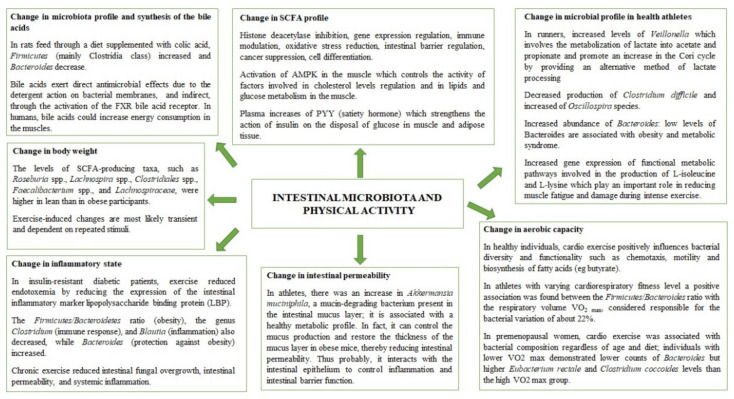
Main effects of diets on gut microbiota.

**Figure 2:**
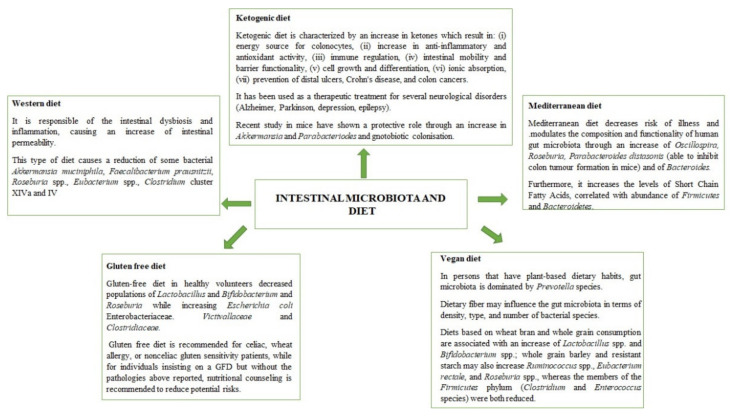
Main effects of physical exercises on gut microbiota.

Future research should also use methodologies to elucidate the effects of exercise on the microbiota in various regions of the gastrointestinal tract, including bacterial species associated with the intestinal mucus layer, although this will likely involve more invasive endoscopic procedures for human studies. Furthermore, it would also be interesting to understand how exercise affects the archaea, fungi, and viruses in the human gut and how it affects intestinal competition and ecological patterns. As a final result of this paper, we propose two mind-maps (Figure 1 and Figure 2), which could help researchers to understand the main effects of diet and physical activity on gut microbiota as a prodromal step to design new intervention studies.

## Data Availability

Not applicable.

## Supplementary Materials

The following supporting information can be downloaded at: https://www.mdpi.com/article/10.3390/nu14122456/s1, Supplementary Material S1. Dietary components and gut microbiota. Supplementary Material S1 Table S1: Effects of fats on gut microbiota. Supplementary Material S1 Table S2: Effects of proteins on gut microbiota. Supplementary Material S1 Table S3: Effects of carbohydrates on gut microbiota [82,83,84,85,86,87,88,89,90,91,92,93,94,95,96,97,98,99,100,101,102,103,104,105,106,107].
