# Supplementary material for: How Diet and Physical Activity Modulate Gut Microbiota: Evidence, and Perspectives
Source: Nutrients. 2022 Jun 14;14(12):2456. doi: 10.3390/nu14122456 (PMC9227967; doi:10.3390/nu14122456)
Supplement: Supplementary file 1 [file nutrients-14-02456-s001.zip › nutrients-1761196-supplementary.pdf]

Supplementary Material 1. Dietary components and gut microbiota

WHO and FAO recommends that daily dietary fat should account 30% of total energy intake, protein should not exceed the 10–15%, and carbohydrate should cover the remaining 55-75%, while a dietary fibre intake is recommended for women around 25 gr/day and 38 gr/day for men [82]. Although these are the recommended proportions, the concentrations of these macronutrients vary according to the diet followed. In addition, studies have shown that differences along with quantity of consumed macronutrients can dramatically influence the human gut microbiota composition and functionality.

S1.1 Fats

Dietary fat is not a homogenous macronutrient, as the functions and structure of different fatty acids can vary remarkably depending on chain length and presence or absence of carbon-carbon double bonds [83]. SFAs have no double bond, MUFAs contain a single double bond, while PUFAs have two or more double bonds, also the double bond can be in a cis or trans configuration. Trans fats are produced industrially by the partial hydrogenation of unsaturated fats or naturally via biohydrogenation in rumen [84]. Some studies have shown that fatty acids exert various effects on the gut microbiota (Table S1).

S1.2 Proteins

Normally, around 25 g of proteins, peptides and free amino acids reach the colon daily [85]. Microbial digestion of these materials in the colon produces a range of end products, like SCFAs, amines, indoles, phenols, hydrogen sulphide, thiols, CO_2 and H_2, where some of them are pivotal for health maintenance and some others are detrimental [86]. Protein quality, quantity, source and processing history, impact on protein digestion and functions, thus they must be careful considered (Table S2).

S1.3 Carbohydrates

Approximately, the daily intake of carbohydrates that entering in the colon is 40 gr [87]. It is important to know that dietary carbohydrates can be divided as resistant starch, non-starch polysaccharides, oligosaccharides, and mono- and disaccharides [88], and like the other macronutrients, the amount and the type of carbohydrate have an impact on the gut microbiota. Resistant starch (RS), which is an important non-digestible carbohydrate "resists" digestion and passes straight through small intestines with minimal absorption. It is divided in four types consumed with diet: Resistant Starch type 1 (RS1), Resistant Starch type 2 (RS2), Resistant Starch type 3 (RS3), and Resistant Starch type 4 [89], as follows: i) RS1: is abundant in whole-grain foods, seeds, and legumes. It has a hard coating that physically protects it from breakdown. Thus, it passes through your body without being absorbed. ii) RS2: is a granular starch. Because of its tightly packed structure, digestive enzymes can't break it down so it resists digestion and absorption. This type of starch is present in green bananas, high-amylose corn, Basmati rice, and raw potatoes. iii) RS3: is also called retrograded starch because it forms when you expose starchy foods, like potatoes, to cold temperatures after cooking them. With cold exposure, a portion of the starch in the food is converted to resistant starch. iv) RS4: is a synthetic form of resistant starch that is chemically modified to make it resistant to digestive enzymes. An example is high-maize resistant starch available at some food markets.

Focusing on fibres, they generally support the growth of several health-promoting genera, like *Bifidobacterium*, *Coprococcus*, *Bacteroides* and *Lachnoclostridium*. Also, SCFAs could be affected by some compounds, for example, propionic acid was increased by arabinogalactin, mucin and xylan but not by inulin or galacto-fructooligosaccharides. Fermentation of mucin produced acetate and propionate, and the highest amount of butyrate favoured the growth of some genera such as *Clostridium*, *Parabacteroides* and *Lachnoclostridium* [90] (Table S3).

Supplementary Material 1 Table S1: Effects of fats on gut microbiota.

Sample	Fats	Effects	Ref.
Mice	Saturated Fats – (SFAs, palm oil)	SFAs induced higher body weight gain and liver triglyceride content compared to diets rich in polyunsaturated fats (olive oil or safflower oil). Furthermore, high-saturated fat diet with palm oil, decreased microbial diversity and increased the Firmicutes/Bacteroidetes ratio, probably due to antimicrobial effects of the saturated fats.	[91]
Mice	SFAs derived from milk's fat	SFAs affected sulphite reducing *Bilophila wadsworthia* that was associated with a pro-inflammatory immune response and increased incidence of colitis in genetically susceptible mice, but not in wild type mice.	[24]
Mice	Milk fat and safflower oil	Increase of *B. wadsworthia* together with a reduction of Tenericutes, a phylum which has been shown to be decreased under inflammatory conditions.	[92]
Mice	Extra virgin olive oil and butter	Highest values of systolic blood pressure in mice fed with butter, positively correlated with the presence of *Desulfovibrio* in faeces, which were notably higher compared to mouse fed with extra virgin olive oil (the inferior levels may be due to the greater presence of polyphenols).<br>In addition, extra virgin olive oil had the lower values of plasmatic insulin (correlated inversely with *Desulfovibrio*) and of leptin (correlated inversely with *Marispirillum*, Sutterellaceae, and *Mucilaginibacter dageonensis* which were particularly higher for extra virgin olive oil).	[93]
Mice	SAFs	Fast increase of hydrogen sulphite-producing bacteria, while these bacteria remained stable in mice fed with omega-6 PUFAs or were reduced in mice fed with omega-3.	[94]
Mice	SAFs	In mice fed for 20 weeks with a diet rich in SAFs a greater abundance of 3 types of sulfidogenic bacteria (*Desulfobulbus*, *Desulfovibrio* and *B. wadsworthia*) compared to mice fed with a low-fat diet, was observed.	[95]
Mice	Partially Hydrogenated Oils (PHOs)	The PHOs showed minimal impact on the mice's gut microbiota and preserved the *Bacteroidetes/Firmicutes* ratio.	[96]
Mice	trans-10, cis-12 Conjugated Linoleic Acid (CLA)	Mice feed with dietary for at least eight weeks resulted in lower proportions of Firmicutes and higher ratios of Bacteroidetes compared with mice which received no supplementation. These results indicate that gut microbiota alterations following long-term supplementation of the trans-10, cis-12 CLA isomer could be harmful to health. Although CLA supplements are commercially available, a fatty acid mixture with equivalent proportions of CLA isomers or probiotics and prebiotics can balance these negative effects.	[97]

Supplementary Material 1 Table S2: Effects of proteins on gut microbiota.

Sample	Proteins	Effects	Ref.
Wistar rats	Diet rich in protein (45% protein, 30% carbohydrate)	Harmful effects on the colonic microbiota compared to a normal protein diet (20% protein, 56% carbohydrate).	[98]
Mice	High protein diet	*E. coli/Shigella*, *Streptococcus*, *Enterococcus* and sulphate-reducing bacteria increased by 5.36, 54.9, 31.3 and 2.59-fold, respectively. Butyrate producers, like *Faecalibacterium prausnitzii* and *Ruminococcus* spp., were decreased respectively by 3.5 and 8.04-fold, while the mucin degrader *Akkermansia muciniphila* (a health promoting bacterium) was not detected in high protein diet group.	[99]
Mice	Soy protein	Increased faecal SCFAs compared to mice fed with red meat, white meat, or casein.	[100]
Mice	Soy protein	Higher relative abundance of *Prevotella* and *Bacteroides.*	[101]
Hamsters	Soy and milk protein	Hamsters fed with the milk protein had a higher relative abundance of *Bacteroidaceae*, *Erysipelotrichacaea* and *Porphyromonadaceae* compared to the hamsters fed with soy.	[102]
Hamsters	Partially hydrolyzed soy protein	A bloom of *Bifidobacteriaceae* and a higher proportion of Clostridiales spp., microbes involved in the maintenance of overall gut function, compared to the milk protein isolate fed group, was observed.	[103]

Supplementary Material 1 Table S3: Effects of carbohydrates on gut microbiota.

Sample	Carbohydrates	Effects	Ref.
Human volunteers	RS3	A diet high in for 10 weeks was found to enhance the growth of *Firmicutes* bacteria related to *Ruminococcus bromii* and *Eubacterium rectale.*	[104]
Human subjects	RS2 and RS4	Different effects of RS2 and RS4 on the composition of the fecal microbiota: in particular, a three week-diet with RS4 reduced Firmicutes and increased Bacteroidetes and Actinobacteria; at the species level, RS4 increased *Bifidobacterium adolescentis* and *Parabacteroides distasonis*. However, data suggest that the response to resistant starches is interindividual and reversible.	[105]
Cohort of twenty young healthy individuals	RS2	RS2 increased faecal butyrate, but the responses to diet were clustered in three groups based on butyrate levels before and during consumption of resistant starch: enhanced, high, and low, also related to a different qualitative composition of gut microbiota. In the enhanced group, higher levels of *B. adolescentis*, *Ruminococcus* spp., and *E. rectale* were found.	[106]
Rats	high fat/high sugar, low fat/high sugar and low fat/low sugar diets	For four weeks, both the high fat/high sugar and low fat/high sugar diets provoked important increases in body fat and body weight compared to the low fat/low sugar diet. The high sugar and high fat diets provoked gut microbiota dysbiosis with a decrease in microbial diversity, an increase in Clostridia and Bacilli and a notable decrease in *Lactobacillus* spp., as well as an increase of Firmicutes/Bacteriodetes ratio. In particular, the low fat/high sugar diet determined an increase in two Proteobacteria members, that are *Bilophila* and *Sutterella*.	[107]
